# Neural activity in the medial parietal area V6A while grasping with or without visual feedback

**DOI:** 10.1038/srep28893

**Published:** 2016-07-06

**Authors:** Rossella Breveglieri, Annalisa Bosco, Claudio Galletti, Lauretta Passarelli, Patrizia Fattori

**Affiliations:** 1 Department of Pharmacy and Biotechnology (FaBiT), University of Bologna, Piazza di Porta San Donato, 2, 40126 Bologna (Italy)

## Abstract

Recent works have reported that grasping movements are controlled not only by the dorsolateral visual stream, as generally thought, but also by the dorsomedial visual stream, and in particular by the medial posterior parietal area V6A. To date, the grasping activity of V6A neurons has been studied only in darkness. Here we studied the effect of visual feedback on grasp-related discharges of V6A neurons while the monkey was preparing and executing the grasping of a handle. We found that V6A grasping activity could be excited or inhibited by visual information. The neural population was divided into Visual, Motor, and Visuomotor cells. The majority of Visual and Visuomotor neurons did not respond to passive observation of the handle, suggesting that vision of action, rather than object vision, is the most effective factor. The present findings highlight the role of the dorsomedial visual stream in integrating visual and motor signals to monitor and correct grasping.

Visual information about the environment, limb movements, and targets of prehension is necessary to guide the hand towards objects to be grasped. This information is essential for planning the action as well as for correcting errors in arm trajectory^1^,^2^,^3^. Area V6A^4^ is a visuomotor area of the dorsomedial visual stream^5^ which is involved typically in the control of arm reaching movements in both monkeys^6^,^7^ and humans^8^,^9^,^10^. Recently, it has been reported that many V6A cells are able to encode the grasping movements, such as those performed to grasp a handle^11^ or other objects of different shapes^12^,^13^. Given the visual nature of V6A^4^,^14^, the grasping activity in these experiments was investigated in darkness, in order to exclude the visual inputs as modulators of the observed grasp-related responses. But what about the influence of light upon this grasping activity? In the present work, we want to assess whether, and to what extent, visual information affects the activity of V6A cells when preparing and executing a grasping action.

For this purpose, we trained monkeys to grasp, in the light and in the dark, a handle with different orientations. We found three categories of neurons: Visual neurons, discharging only in the light, Motor neurons, discharging equally in the dark and the light, and Visuomotor neurons, the most represented type of neurons in this area, discharging both in the dark and the light, but differently under the two visual conditions. Since the arm movement-related activity in the light reflects visual and proprioceptive afferences as well as motor efferent copies, whereas the activity in the dark reflects only proprioceptive and motor efferent feedbacks, the results suggest that Visual and Motor neurons receive only visual or proprioceptive/motor efferent copy signals, respectively, while Visuomotor neurons incorporate both types of signals and likely integrate them in the guidance of prehension. The present results help in understanding the functional role that different sensorimotor modulations play in the activity of V6A grasping cells.

## Results

**Figure 1:**
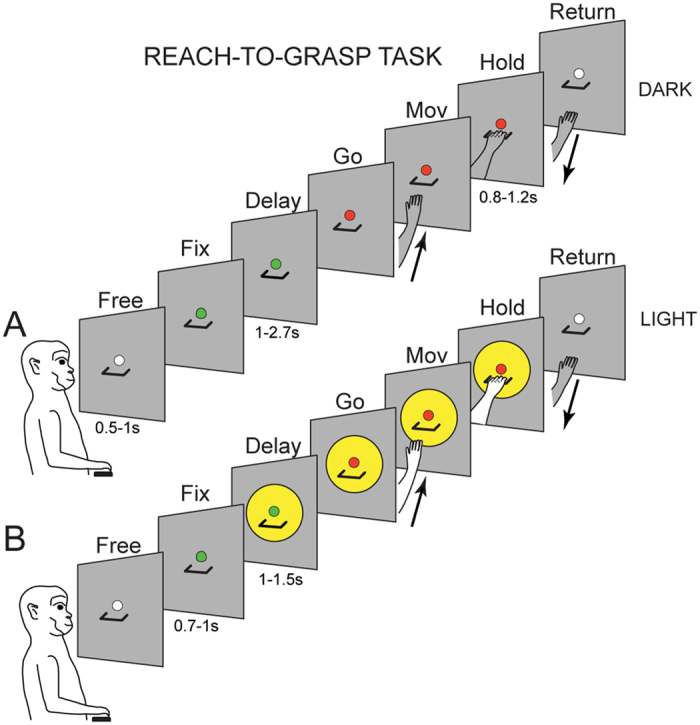
Schematic representation of the instructed-delay reach-to-grasp task. (**A**,**B**) Time course and time epochs in the reach-to-grasp task in the dark (**A**) and in the light (**B**).

To understand the contribution of visual information on the grasp-related activity of V6A cells, we employed a task where monkeys reached and grasped, in the dark (Fig. 1A) and in the light (Fig. 1B), a handle that was located in a constant spatial position and could have different orientations. In the light (Fig. 1B), the handle and the working-space were illuminated during movement preparation, execution, and handle pulling. Video inspections of the hand movements confirmed that monkeys adopted the same grip (finger prehension) when grasping horizontal and vertical handles, though with different wrist orientation. Movement time for handle grasping in the dark was significantly higher than in the light (mean value in the dark: 333 ± 121 ms; mean value in the light: 301 ± 102 ms, Student’s t-test, p < 0.05).

A total of 262 neurons were recorded from area V6A in three monkeys while the animals performed the Reach-to-grasp task in two visual conditions, light and dark. Results were consistent between animals (Chi-square test, p > 0.05), thus they will be presented jointly. Area V6A was identified during recording sessions on the basis of the functional properties of its neurons according to previous studies^4^,^15^. Histological controls allowed us to check whether recording sites were within the limits of area V6A, in the anterior wall of the parieto-occipital sulcus, according to the cyto- and myeloarchitecture of this area described in other reports^14^,^16^.

We examined the neural discharge during movement preparation (epoch PLAN, the last 500 ms before the instruction signal to reach-to-grasp the handle), grasping execution (epoch MOV, from 200 ms before the movement onset to movement end) and handle pulling (epoch HOLD, from the onset of handle pulling to 200 ms before the return movement onset). We considered as baseline activity the neural discharge at the beginning of the trial, before fixation onset (epoch FREE). By comparing neural activity during epochs PLAN, MOV, HOLD versus FREE, 243 out of 262 recorded cells (93%) were task related (i.e., modulated in at least one of the epochs and in at least one visual condition; Student’s t-test corrected for multiple comparisons, p < 0.02).

**Figure 2:**
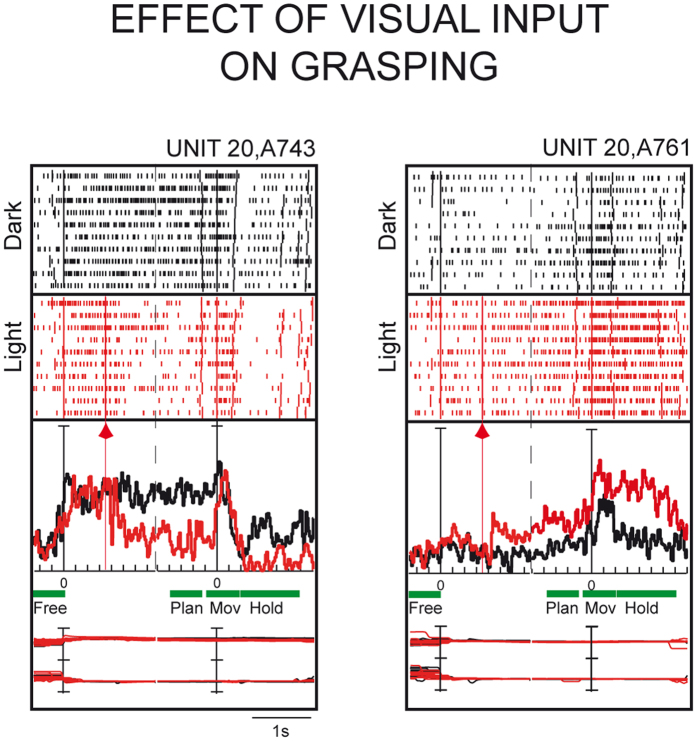
Effect of visual information on neuronal discharge. Examples of cells whose grasping activity is modulated by the visual feedback. Left, cell inhibited by the visual input during PLAN and HOLD. Right, cell activated by the visual input during PLAN, MOV and HOLD. Different colors indicate various visual conditions, for which spike rasters (on top), averaged firing rates (at the middle) and eye traces (bottom) are shown individually. The two vertical lines at zero indicate the beginning of ocular fixation and the release of the home button (movement onset), on which all trials are aligned. Arrowheads indicate the illumination onset of the handle in the trials in the light. Below the averaged firing rates, functional epoch durations are depicted: Scale: vertical bar on histograms, 60 (left), 70 (right) spikes/s; eye traces: 60 degrees per division. Statistical values for the cell to the left (Student’s t-test): FREE vs PLAN in the dark, p = 1.54e-05, in the light, p = 0.57; FREE vs MOV in the dark, p = 3.73e-0.06, in the light p = 3.58e-004; FREE vs HOLD in the dark, p = 0.012, in the light, p = 0.048). Statistical values for the cell to the right (Student’s t-test): FREE vs PLAN in the dark, p = 0.43, in the light, p = 0.0024; FREE vs MOV in the dark, p = 0.0024, in the light p = 1.73e-007; FREE vs HOLD in the dark, p = 0.0243, in the light, p = 1.86e-007).

Figure 2 shows two examples of task-related cells modulated differently by visual input during grasping. The neuron on the left in Fig. 2 was modulated during PLAN, MOV, and HOLD. In darkness, the cell’s discharge suddenly increased as soon as the animal began fixation, and remained high and quite constant up to the movement onset. After a small peak of activity during the movement time, the activity decreased just after the grasping of the handle, rising again above the baseline level during HOLD. In the light, the cell’s discharge suddenly increased as soon as the animal began fixation, as in darkness, but it was inhibited when the handle was illuminated, and kept on being inhibited during PLAN and HOLD, but not during the execution of reach-to-grasp movement (MOV). Also the neuron on the right in Fig. 2 was modulated in all three epochs. In darkness, there was a gradual increase of discharge in PLAN, a transient increase of activity during MOV, and a reduction of discharge rate during HOLD, though the activity remained above the baseline level. In the light, the cell suddenly increased the activity at the onset of illumination, then the discharge rate remained higher than in darkness in all the epochs of the grasping task.

To quantify how strongly the grasping activity of individual neurons was modulated by the visual input, we used a visual index (VI; see Methods) that takes into account the strongest discharges in the light and in the dark. The value of VI ranges from −1 to 1 and indicates whether and how much the mean activity in the light is higher (positive value) or lower (negative value) with respect to that in darkness. Half of the population was excited by the visual input (119/243 in PLAN, 129/243 in MOV; 130/243 in HOLD), showing VI > 0, and half (124/243 in PLAN, 114/243 in MOV; 113/243 in HOLD) was inhibited (VI < 0).

**Figure 3:**
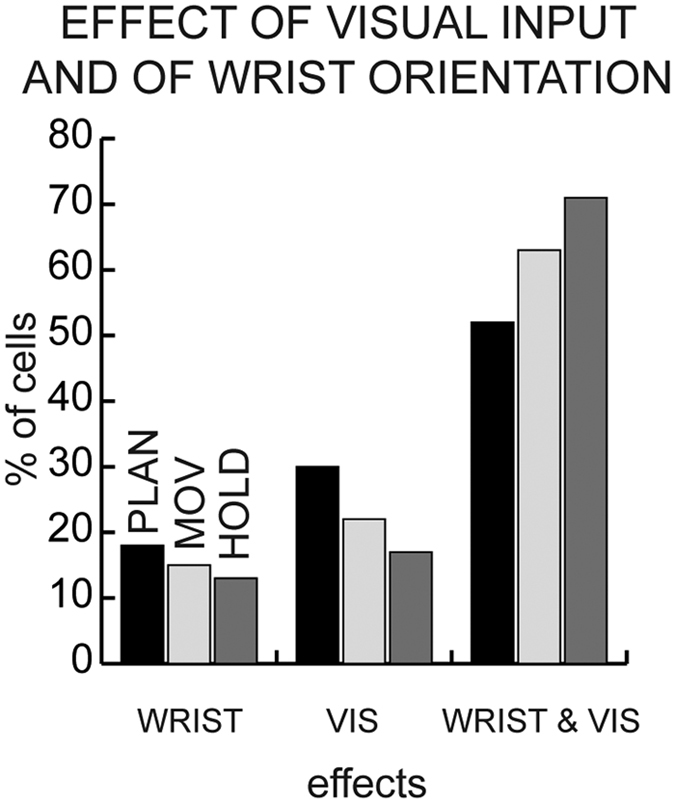
Distribution of the incidence of significant effects modulating V6A cells during the time-course of the task. Histograms show the results of two-way ANOVA (VIS × WRIST, p < 0.05) as incidence of modulated cells during PLAN, MOV and HOLD. The results are shown with respect to effect complexity, that is, from left to right, the main effects and the effect of both factors. Numbers of modulated cells: effect of VIS: N = 52 (PLAN), N = 46 (MOV), N = 35 (HOLD); effect of WRIST: N = 31 (PLAN), N = 31 (MOV), N = 26 (HOLD); effect of both VIS and WRIST: N = 90 (PLAN), N = 131 (MOV), N = 146 (HOLD); no effect (not shown in the figure): N = 70 (PLAN), N = 35 (MOV), N = 36 (HOLD).

We investigated the effect of visual condition (VIS) and of wrist orientation (WRIST) on task-related cells. As shown in Fig. 3, WRIST and VIS influenced a minority of V6A cells (2-ways ANOVA, factors: wrist orientation, visual condition, p < 0.05), with cells modulated by visual background more numerous, although not significantly, than those modulated by wrist orientation (about 20% VIS *versus* about 15% WRIST). Most of the cells were influenced by both WRIST&VIS (Fig. 3), with about 50% of cells modulated by both of these factors during PLAN, and 65–70% of cells during MOV and HOLD.

Figure 3 also shows that V6A cells were modulated differently during the time course of the task. While pure WRIST and VIS modulations decreased but did not change significantly during the trial (Chi-square test, p > 0.05), there was a significant increase of WRIST&VIS modulation during the task (Chi-square test, p < 0.05).

Overall, Fig. 3 shows that the effect of WRIST&VIS in combination was prominent in V6A, and affected the activity of neurons especially during reach-to-grasp execution and object holding.

**Figure 4:**
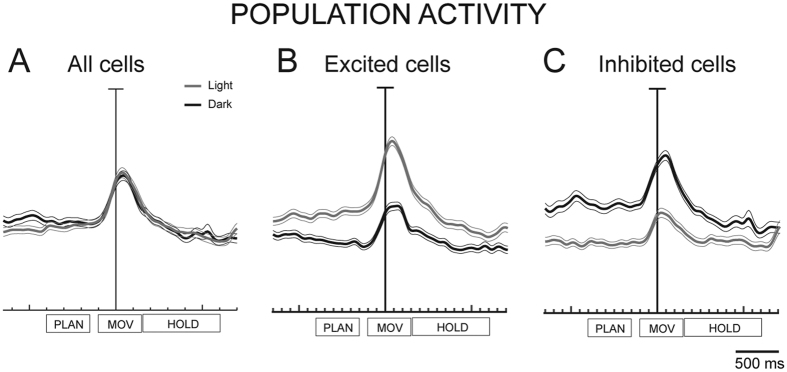
Effect of vision on grasping activity of V6A cells: population discharge. (**A**) Average SDF (thick line) of the cells modulated by visual information. Grey: neural activity recorded in the light; black line indicates cells tested in the dark. Each cell was taken into account twice: once for the preferred orientation in the light, and another for the preferred orientation in the dark. The thin lines indicate the variability band (SEM). (**B,C**) Average SDF of cells whose activity in the light is higher than in the dark (excited cells, B, N = 119) and of cells whose activity in the dark is higher than in the light (inhibited cells, C, N = 116). The activity of cells in each plot was aligned on the onset of arm movement. Rectangles below each plot indicate the time epochs analyzed (PLAN, MOV, HOLD).

The cumulative spike density functions (SDFs) of the cells modulated by visual information (Fig. 4A) show that, at population level, the activity in the light was not different from the one in the dark in PLAN, MOV and HOLD (permutation test, p < 0.05). At first glance this may appear strange, as this cumulative activity comes from cells significantly modulated by visual information. However, the analysis of the VI indicated that half of the grasp-related cells were excited in the light (see Fig. 4B) and the other half inhibited (see Fig. 4C). It is evident that in the excited cells (Fig. 4B) the activity was on average higher in the light than in the dark in all action epochs (permutation tests, *p* < 0.05), whereas in the inhibited cells (Fig. 4C) the activity in the dark was higher in all epochs (permutation test, *p* < 0.05). The absence of influence of visual feedback on V6A if the neuronal population is taken as a whole (see Fig. 4A) has to be considered when designing fMRI experiments: on the basis of the present results we expect no BOLD activation in V6A when contrasting grasp-related activations in the light and in the dark.

**Figure 5:**
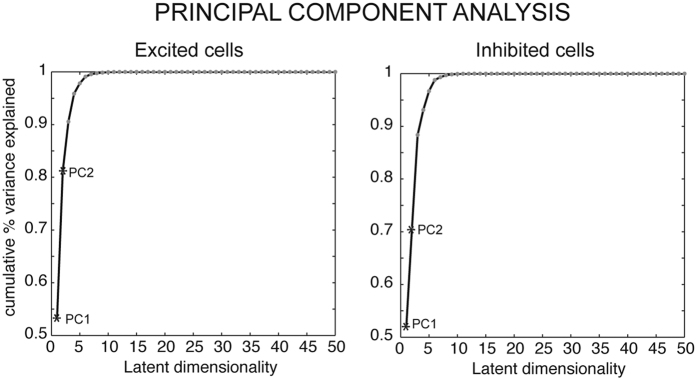
Principal components analysis of grasping movements. Each plot represents cumulative sum distributions of the relative weights (in percentages, y values) of the eigenvectors of the principal components (PC). PC1, first principal component, PC2, second principal component. Latent dimensionality: PCs.

To further determine neural activity patterns that may have not been captured by the above statistical analyses, we performed a principal component analysis (PCA) on the data. This analysis simplifies the intrinsic complexity of the neural discharges and can provide further insight for data interpretation because each eigenvalue represents the weight of the corresponding component of the data, i.e., the amount of variance explained. The relative weights of the eigenvectors, which exemplify their capacity of representing the whole dataset, were obtained normalizing the eigenvalues and are shown in Fig. 5. In both the excited and the inhibited cells, the weight of the first principal component was around 50% (52% for excited cells, 53% for inhibited cells). In the excited cells (Fig. 5, left), the sum of the eigenvalues of the first and second principal components was close to 80% (i.e. the first two extracted components accounted for 80% of the data variability), whereas in the inhibited cells (Fig. 5, right), on the contrary, the sum of the eigenvalues of the first two principal components reached only about 70%. This difference suggests that the population of the inhibited cells is more heterogeneous than the excited ones, and that the neural processes occurring in inhibited cells are more complex than those in the excited ones. When performing PCA for each epoch (DELAY, MOV, HOLD) independently, we observed the same higher eterogeneity of inhibited cells with respect to the excited ones. Moreover, we observed that, going from DELAY to HOLD more components were needed to account for the same amount of data variability. This means that there was an increase of complexity in the population discharge in the time-course of the task.

### Effect of visual input on wrist orientation tuning

**Figure 6:**
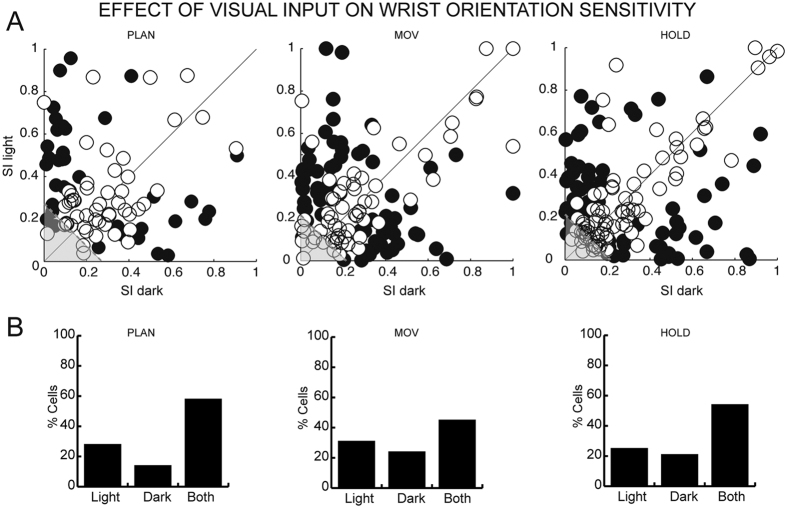
Differences in sharpness of orientation tuning of V6A cells in the two visual conditions. (**A**) Distribution of sensitivity indices of cells calculated for PLAN, MOV, and HOLD in dark and light conditions. Each point represents one neuron. The filled circles indicate neurons whose bootstrap-estimated confidence intervals do not cross the diagonal (‘Light’ or ‘Dark’ cells). Empty circles indicate neurons whose bootstrap-estimated confidence intervals cross the diagonal (‘Both’ cells). The dashed grey line indicates the level below which differences could be due to noise. Noise line equations: PLAN: x + y = 0.27; MOV: x + y = 0.21; HOLD: x + y = 0.21. Data in the grey area below the grey lines are below the noise level. (**B**) Distributions of ‘Light’, ‘Dark’, and ‘Both’ cells in the three epochs considered. The majority of our neural population belongs to ‘Both’ cells, indicating that visual input did not alter significantly the sharpness of the orientation tuning.

As reported above, a high number of V6A cells were modulated by both vision and wrist orientation. To study the influence of visual input on wrist orientation tuning, we measured the sensitivity of each neuron to the wrist orientation in each of the 2 visual conditions tested by calculating a specific sensitivity index (SI; see Methods). We performed this analysis on neurons sensitive to both wrist orientation and visual condition (for PLAN: N = 90 cells; for MOV: N = 131 cells; for HOLD: N = 146 cells). To compare the SIs of a cell in different visual conditions, confidence intervals on the preference indices were estimated using a bootstrap test. The data we obtained are summarized in Fig. 6A. Each black dot represents a cell significantly sensitive in one of the two visual conditions (bootstrap test, 10,000 iterations, p < 0.05). Cells characterized by a wrist tuning independent of the visual condition (whose bootstrap-estimated confidence interval crosses the diagonal) are represented as white dots. Symbols located above the diagonal are neurons with wrist sensitivity significantly stronger in the light. A sensitivity stronger in darkness occurs for cells with symbols located below the diagonal. This analysis shows first that the strength of modulation of grasping activity in the light and in the dark varied across cells. In addition, the analysis shows that a minority of neurons were more sensitive to wrist orientation in the light or in the dark (see column ‘Light’ and ‘Dark’ in Fig. 6B), while about half of the neurons were similarly sensitive in the two visual conditions (see column ‘Both’ in Fig. 6B), without significant differences among the three epochs (Chi-square test, p > 0.05).

### Visual, Motor, and Visuomotor neurons

Considering the widespread use of the classification of grasping cells in Visual, Motor, and Visuomotor neurons after the seminal work of Sakata and colleagues^17^, we divided the task-related neurons accordingly (see Methods for details). *Visual* neurons discharged during reach-to-grasp execution but only in the light (visual response), *Motor* neurons discharged during reach-to-grasp execution in the light and in the dark with the same strength (motor response), *Visuomotor* neurons discharged during reach-to-grasp execution in the light and/or in the dark with a different strength (visuomotor response).

**Figure 7:**
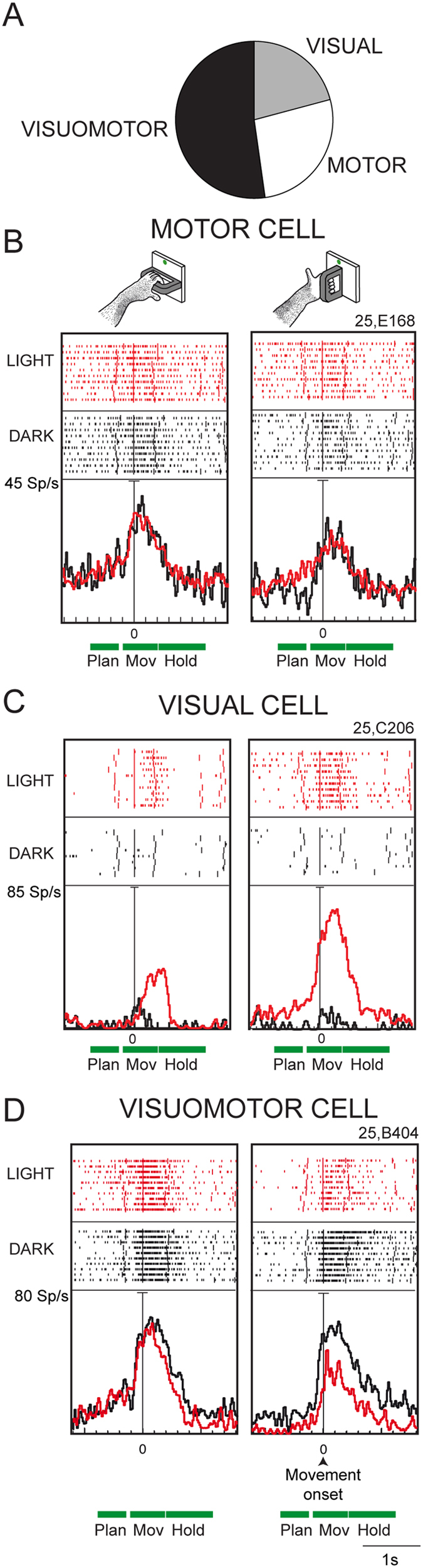
Visual, Motor and Visuomotor neurons. (**A**) Pie-chart showing the incidence of the three categories of cells; (**B–D**) Examples of single neurons. (**B**) Motor cell. Horizontal handle grasping trials are shown on the left panel and vertical handle grasping on the right panel. (**C**) Visual cell. (**D**) Visuomotor cell. Responses are aligned on movement onset. Scale: vertical bar on histograms, 45 (**B**), 85 (**C**), 80 (**D**) spikes/s. Other conventions as in Fig. 2.

A minority of cells in V6A were Visual (N = 42, 21%), or Motor (N = 54, 27%), whereas the majority were Visuomotor (N = 103, 52%; see Fig. 7A).

Figure 7B–D shows examples of the three different types of neurons. Fig. 7B shows the grasping activity of a Motor neuron in the light and in the dark. The cell was modulated strongly by reach-to grasp movement in both visual conditions, with wrist orientation significantly affecting cell activity, with a preference for grasping the handle with the wrist pronated (horizontal handle). The vision of the handle and of the moving arm/hand did not affect significantly cell activity.

Figure 7C shows the discharge of a Visual neuron. In the light, this cell showed a strong discharge in MOV, that was modulated strongly by wrist orientation, with a preference for the vertical handle, while in the dark both grasping discharge and task modulation almost disappeared. The conclusion is that the strong response in the light was likely due to the vision of the hand grasping the handle.

Figure 7D shows one example of Visuomotor neuron, the most representative type of cells in V6A (see also examples of this type of neurons in Fig. 2). This cell was modulated during PLAN and MOV and was well activated by grasping both in the light and in the dark. However, in the light, the wrist orientation significantly modulated the cell, whereas in the dark the grasping discharge remained more or less the same regardless of wrist orientation.

The present results indicate that V6A contains a minority of Visual- and Motor cells, that is cells modulated only by the visual stimulus or by the reach-to-grasp motor act, respectively, and a majority of Visuomotor cells, that is cells modulated by both factors. Visual neurons likely encode visual information about the object (grasped, or to be grasped) and/or about the reach-to-grasp action. Motor neurons likely reflect the efference copy of motor signal and/or the proprioceptive feedback of action. Visuomotor neurons likely integrate visual and motor-related signals about the whole act of prehension.

### Visual control task

A Visual control task was performed with the same structure as the reach-to-grasp task in the light, but with the animal not allowed to perform any arm movement, so the handle was not the target of a grasping action. This Visual control task was performed on single cells that were already tested with the Reach-to-grasp task, to ascertain whether the activity of Visual and Visuomotor neurons during grasping execution in the light reflects the vision of the object to be grasped, or the vision of the grasping hand, or both. Our hypothesis was that if Visual or Visuomotor neurons did not show any activity during passive handle observation, the grasping activity in the light had to be ascribed to the vision of the hand performing the grasping action. These neurons would be similar to the ‘non-object’ type neurons of AIP^17^,^18^,^19^, or to similar neurons recently found in parietal and frontal areas^20^,^21^,^22^. If, on the contrary, Visual and Visuomotor neurons were responsive to passive handle observation, and this activity was similar to that observed during grasping execution in the light, the conclusion would be that the cell activity was (only) influenced by the visual attributes of the object to be grasped. This category of cells would correspond to the ‘object-type’ neurons found in area AIP^17^,^18^,^19^. Finally, if Visual and Visuomotor cells discharged to both passive vision of the handle and execution of the action in the light with different strength, these cells would be sensitive to both object vision and action vision. Responses to the passive presentation of the handle when no reach-to-grasp movements were required were evaluated in a subset of 100 V6A neurons from 2 animals. Out of the 100 cells that underwent the Visual control task, 23 proved to be Visual cells, 48 Visuomotor, and the remaining 29 Motor cells. This distribution of cell types reflected what was found in the entire V6A population we tested.

**Figure 8:**
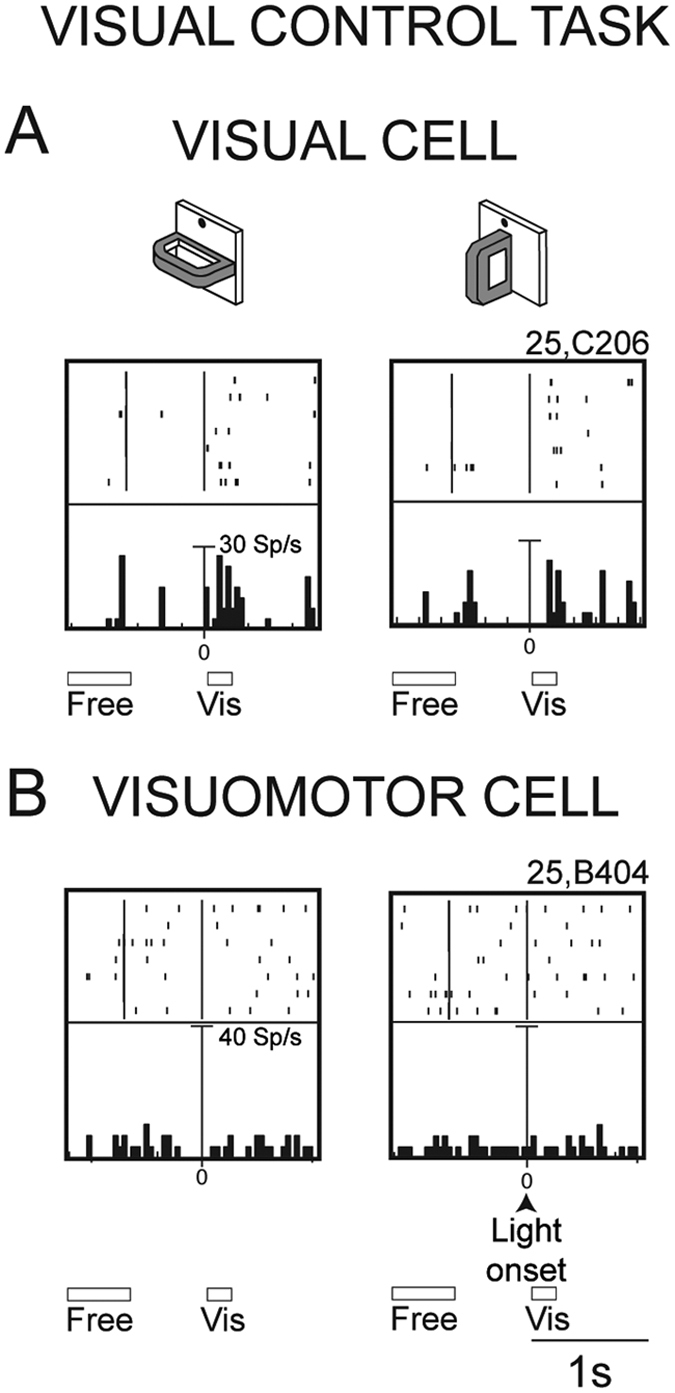
Responses in the Visual control task. Discharges for the vision of the handle of the Visual (**A**) and Visuomotor (**B**) cells shown in Fig. 7 (**C**,**D**), respectively. Responses are aligned on object illumination. (**A**) The cell of Fig. 7C is also activated for the vision of the handle, even if the discharge for the vision of the grasping action (Fig. 7C) is significantly higher (Student’s t-test, p < 0.05). (**B**) The cell of Fig. 7D (visual cell) is not modulated by the vision of the handle (Student’s t-test, p > 0.05). Scale: vertical bar on histograms, 30 (**A**), 40 (**B**) spikes/s. Other conventions as in Figs 2 and 7.

Figure 8 shows the responses to handle observation of the Visual (Fig. 8A) and Visuomotor (Fig. 8B) cells shown in Fig. 7. The Visual cell showed a weak response to handle presentation; the Visuomotor cell did not respond at all to the vision of the handle. Among the V6A grasping cells that showed a significant visual response to handle presentation (17% Visual cells and 35% Visuomotor cells), more than half (67%) showed grasp-related responses in the light significantly higher than the passive visual responses observed in Visual Control task (Student’s t-test, p < 0.05). This was the case for the cell shown in Figs 7C and 8A, that displayed a very weak though significant visual response to the passive handle observation and a much stronger response during grasping observation in the light. This kind of neuron is slightly sensitive, if at all, to the vision of the object, but strongly encodes the vision of grasping action.

The majority of both Visual (83%) and Visuomotor (65%) neurons did not respond significantly to the passive observation of the handle (Student’s t-test, p < 0.05). These cells were activated by grasping execution, but discharged differently to the execution in the light and in the dark. In some cases the discharge frequency during execution in the light was higher, in others lower, as in the example of Fig. 7D. In any case, these neurons would encode the vision of action unfolding, rather than the “passive” vision of graspable object. It should be noted, however, that the responses in the Visual control task were collected in experimental conditions (grasping execution and object observation) that required different levels of attention by the animal, and the attentional level could be an additional factor responsible for cell modulation, as already documented for V6A neurons^23^.

## Discussion

We recorded spiking activity from area V6A while monkeys grasped a handle that could have different orientations while maintaining a fixed gaze position. Grasping was planned and executed either in darkness or in the light. The constant position in space of the handle and of the fixation point precluded any possible modulation of neuronal activity related to the direction of arm movement, direction of gaze, or relative position between the two, all factors strongly affecting neural discharge in V6A^7^,^24^,^25^,^26^. And since, as checked by video inspections, the animal always grasped the handle with the same type of grip (finger prehension), nor could the grip type, another factor known to affect V6A grasping neurons^12^, account for possible differences in neuronal discharge.

We found that the activity of about 80% of V6A cells is influenced by visual information (VIS + WRIST&VIS cells, Figs 3 and 4). This result is in agreement with the incidence of visual cells in V6A^4^. These visual cells, often very sensitive to the orientation of visual stimuli^15^, could provide critical visual information to the motor structures that control arm movement and hand orientation during grasping (see also^5^). In good agreement with this hypothesis, V6A is connected directly with premotor area F2^27^,^28^,^29^, which is known to control arm movement and prehension^30^.

Though, as recalled above, visual information influenced the grasp-related discharge in the large majority of V6A cells, a small group of V6A grasping cells (WRIST cells, Fig. 3; about 10%) was not influenced by visual input. The functional role of these cells could be to encode/monitor the action of prehension irrespective of visual information, presumably relying on somatosensory or corollary motor signals that have been demonstrated to affect V6A neurons^7^,^31^.

We found that in many V6A cells the visual background influenced neuronal activity also during grasping preparation besides execution. Note that this effect cannot be ascribed to the visual stimulation produced by the movement of the arm, because during preparation the arm is motionless and outside the animal’s field of view. During this period the activity could be related to the anticipation of the consequences of movement, i.e. to a feedforward signal for the control of arm movement^32^. It is well known that a feedforward control is very useful when motor control strategies must be conceived, in particular before incoming sensory information is used to correct movements^33^,^34^,^35^.

The present results describe an influence of the visual input on *grasping* activity very similar to the effect reported in this same area on *reaching* activity^36^. However, a comparison of the results of the PCA reported here for grasping with those reported in the same area for reaching^36^ reveals that when a reaching movement is performed, the weight of the first principal component is higher (80%, for reaching, see Fig. 10 of 36) than during grasping (50%, see current results). This difference suggests that during reaching, the majority of V6A neurons have a predictable response, whereas during grasping V6A population is more heterogeneous, less synergic in its discharge. This in turn can suggest a simpler involvement of V6A in reaching (or other directional movements) than in movements requiring different wrist orientation.

According to the present results, half of the V6A grasping cells are excited by visual information and half are inhibited. While the neuronal excitation was certainly expected, given that about 60% of V6A cells are sensitive to visual stimulation^4^, the inhibition was less expected, in particular with such a high incidence. However, a similar apparently odd inhibition was already observed in V6A during reach-to-point arm movements^36^, and in other cortical areas during grasping movements (F5 and F1^20^; F2^30^; SII/pIC^37^; early visual cortical areas^38^). It is worth noting that, instead of an inhibition by visual input, the observed phenomenon could be the result of a lack of excitation caused by other factors related to the absence of visual feedback, such as the greater efforts required by grasping objects in the dark^39^,^40^,^41^,^42^. Interestingly, it has been reported that the level of attention influences the activity of single V6A cells^15^,^23^. Therefore, the inhibition while preparing and executing the grasping in the light may indeed be a lack of the neural excitation evoked by the higher level of attention required in darkness. Moreover, the sensory consequences of an action are more represented when the action is performed in the dark^38^. For instance, grasping an object in the dark is much more difficult than doing it in the light, and requires an increased level of alertness.

The stronger discharge in the dark during grasping execution could also be related to corrections of finger posture ensuring proprioceptive, tactile, and force feedbacks following hand-object contact^43^,^44^, or to signaling hand position in the peripersonal space^45^. During grasp planning, the stronger discharge in the dark may also reflect the memory of the object to be grasped, in particular its orientation, that in the dark must be held in the memory up to grasp completion. Note that the neuron shown on the left in Fig. 2 perfectly matches this last consideration, showing, in darkness, a high and constant frequency of discharge from the onset of fixation to grasp completion. To summarize, it seems that the neural processes occurring in inhibited cells are more complex than in excited ones, and this is confirmed by the PCA results (Fig. 5).

Finally, it is worthwhile noting that the control of wrist movements in the light relies particularly on online correction mechanisms^46^,^47^,^48^,^49^,^50^,^51^,^52^,^53^, whereas in the absence of visual information it mainly relies on movement planning^46^,^52^,^53^. Hence, the excitations and inhibitions that we observed might be the result of the interplay between planning and online control exerted by V6A in the control of prehension.

The present results demonstrate that V6A contains a minority of Visual and of Motor cells, and a majority of Visuomotor cells. We also observed that the majority of Visual and Visuomotor cells did not respond to passive handle observation, and the remaining cells responded more strongly to the vision of grasping action than to the vision of handle. This type of cells modulated by the vision of hand configuration during grasping were also found in other parietal^19^,^21^,^22^ and frontal^20^ areas, and highlight the role of visual information in monitoring grasping actions.

An influence of visual information on grasping activity similar to that observed in V6A was reported for neurons of area AIP, a posterior parietal area known to be involved in the control of grasping^17^ and recently found to be directly and reciprocally connected with V6A^27^,^54^. The so-called *Visual-dominant* neurons and *Visual-and-motor* neurons of area AIP increased their activity when the monkey observed its own grasping action in full light conditions^17^,^19^. It was suggested for AIP that the activity observed in the light was related to the vision of the grasping hand or to the combined view of the object and the correspondingly selected hand grip. The same functional interpretation could be suggested for the Visual- and Visuomotor cells of V6A, particularly for those that are insensitive to the passive vision of the handle (Figs 7D and 8B), or are modulated by both handle vision and vision of grasping action (Figs 7C and 8A).

The incidence of Visual, Motor, and Visuomotor neurons in areas V6A and AIP is summarized in Table 1. The distribution of the three types of cells in the two areas is statistically different (Chi-square test, p < 0.05), with V6A containing more Visuomotor neurons than AIP, and AIP more Visual neurons than V6A. Note also that about 90% of AIP neurons were reported to be sensitive to simple visual stimuli, like fragments of shapes^55^, whereas only about 30% of V6A neurons were sensitive to simple visual stimuli^4^,^14^. These differences between the two areas likely reflect a different functional role: AIP seems to be involved in both object recognition and visual monitoring of grasping, whereas V6A seems to be more involved in the visual guidance of reach-to-grasp movements. However, some caution is needed in accepting these interpretations, as the observed differences can be caused by different kinds and numbers of objects used in this and Sakata’s studies^17^,^18^,^19^. In fact, whereas in the present study only one object was tested (a handle), in AIP different objects of different shapes were employed, thus eliciting different types of grips. Further experiments are needed to check for differences between AIP and V6A and therefore to suggest differential functional roles for them. A tempting speculation is that the two areas continuously update and control the configuration and orientation of the hand as it approaches the object to be grasped, but with a different weight of sensory and motor-related signals^52^,^53^. It could also be that the two areas are involved in the control of grasping with a different timing, with V6A more involved in fast, broad control and AIP in the slow, finer control^5^,^56^,^57^. Another difference between V6A and AIP is the lack, in AIP, of the neural inhibition for grasps in the light observed in V6A (see Figs 2 and 4). This could indicate a higher influence of attentional modulations in V6A with a deeper involvement in online control of movement. Apart from these speculations, further experiments are needed to check the different contributions of V6A and AIP, and more generally the dorsomedial and dorsolateral visual streams in the control of grasping.

## Methods

Experiments were carried out in accordance with National laws on care and use of laboratory animals, and with the European Communities Council Directive of 22^nd^ September 2010 (2010/63/EU) and approved by the Bioethics Committee of the University of Bologna.

Three trained male *Macaca fascicularis* weighing 3.1 (case A), 3.8 (case B) and 3.9 (case C) kg sat in a primate chair and were trained to perform a Reach-to-grasp task and a Visual control task under controlled conditions. After training completion, the head-restraint system and the recording chamber were surgically implanted in asepsis and under general anesthesia (sodium thiopenthal, 8 mg/kg/h, *i.v*.) following the procedures reported in Galletti *et al*.^58^. Adequate measures were taken to minimize pain or discomfort. A full program of postoperative analgesia (ketorolac trometazyn, 1 mg/kg *i.m.* immediately after surgery, and 1.6 mg/kg *i.m.* on the following days) and antibiotic care (Ritardomicina^®^ (benzatinic benzylpenicillin + dihydrostreptomycin + streptomycin) 1–1.5 ml/10 kg every 5–6 days) followed the surgery.

Single neurons were extracellularly recorded from area V6A in the anterior bank of the parieto-occipital sulcus. We performed single microelectrode penetrations using home-made glass-coated metal microelectrodes (for animals A and B), and multiple electrode penetrations using a 5 channel multielectrode recording minimatrix (Thomas Recording, GMbH, Giessen, Germany, for animals B and C). The electrode signals were amplified (at a gain of 10,000) and filtered (bandpass between 0.5 and 5 kHz). Action potentials in each channel were isolated with a dual time-amplitude window discriminator (DDIS-1, Bak electronics, Mount Airy, MD, USA) or with a waveform discriminator (Multi Spike Detector, Alpha Omega Engineering, Nazareth, Israel). The recording procedures used for one monkey are similar to those reported in Galletti *et al*.^58^. Briefly, spike times were sampled at 1 KHz, eye movements were recorded simultaneously using an infrared oculometer (Dr. Bouis, Karlsruhe, Germany) and sampled at 100 Hz. The recording procedures for the other monkeys were slightly different and are described in detail in Kutz *et al*.^59^. Briefly, spikes were sampled at 100 KHz and eye position was recorded simultaneously at 100 Hz with a camera-based eye tracker (ISCAN). In all cases eye position was controlled by an electronic window (5 × 5 degrees) centered on the fixation target. Behavioral events were recorded with a resolution of 1 ms.

Monkeys’ arm movements were video-monitored continuously by means of miniature, infrared-illumination-sensitive videocameras. The grip used during reach-to-grasp task in the different handle orientations and visual conditions was estimated using video images at 25 frames/s.

The histological reconstruction of recording sites was performed as described in other works^4^,^14^. Briefly, electrode tracks and location of each recording site were reconstructed on histological sections of the brain on the basis of marking lesions and several other cues, such as the coordinates of penetrations within the recording chamber, the kind of cortical areas passed through before reaching the anterior bank of the parieto-occipital sulcus, and the distance of the recording site from the surface of the hemisphere (for a detailed description of attribution of neural recordings to V6A see ref. 4).

### Behavioral tasks

#### Reach-to-grasp task

Our experiment was conceived to evaluate the contribution of visual environment on the cell’s discharge during grasping movements. For this purpose, we tested the same V6A cell while the monkey performed instructed-delay reach-to-grasp movements towards a handle (aluminium made, thickness 5 mm, width 34 mm, depth 13 mm; gap dimensions: 5 × 28 × 11 mm) located in a constant spatial position on a frontal panel, 22.5 cm from the monkey’s eyes, at 2.2 cm under the straight-ahead visual fixation point, while the gaze was stable on it. The monkey performed arm movements in a Reach-to-grasp task (Fig. 1) with the contralateral arm and with the head restrained.

The reach-to-grasp movement was performed in the light and in the dark: in the dark (Fig. 1A) the monkey could only see the fixation point, whereas in the light (Fig. 1B) two white light-emitting diodes (LEDs) illuminated a circular area (diameter 8 cm) centred on the handle, so that the monkey could see the handle and the arm movement (hand approaching the handle and its grasping), together with the fixation point. Because it was a well-practised action that the monkey performed routinely, it became automatized and it is well known that automatized actions are typically controlled by the dorsal stream^60^.

The handle could have 2 different orientations (horizontal, vertical), so the monkey approached and grasped it with different hand orientations: hand pronated and half pronated, respectively. In both cases the monkeys grasped the handle with the same grip: finger prehension.

As shown in Fig. 1, reach-to-grasp movements started from a button (‘home’ button, 2.5 cm in diameter) placed outside the animal’s field of view, 5 cm in front of the chest, on the animal’s midsagittal line. Reaching movements transported the hand from the button to the handle, positioned at a comfortable arm-length distance.

The reach-to-grasp task in the dark, illustrated in Fig. 1A, had the following time sequence: the trial began when the monkey pressed the home button in complete darkness. After button pressing, the animal awaited instructions in darkness (*Free*). It was free to look around and was not required to perform any eye or arm movement. After 0.5–1 s the fixation LED lit up green and the monkey was required to fixate it waiting for the LED change in color without performing any eye or arm movement (*Fix*). After a delay period of 1–2.7 s (*Delay*), the LED color changed (from green to red). This was the go-signal for the monkey to release the button and perform a reach-to-grasp movement (*Mov*) to reach and grasp the handle, pull it, and maintain it pulled (*Hold*) till the LED switched off (after 0.8–1.2 s). The LED switch-off cued the monkey to release the handle (*Return*) and to press the home-button again. Home-button pressing ended the trial, allowed monkey reward, and started another trial (*Free*).

In the dark condition (Fig. 1A), the animal could only see the fixation LED. The brightness of the LED was reduced so that it was barely visible during the task: standing by the monkey, the experimenter could not see the handle or the monkey’s hand moving in the peripersonal space, even in dark-adapted conditions. Thus, in this condition, we can exclude that cell modulation was the consequence of visual feedback evoked by the movement of the arm in the visual field.

In the light condition (Fig. 1B), the fixation LED, the handle, and the entire grasping movement were visible in the epochs considered. The task was the same as in the dark, except for the fact that after a fixation period of 0.7–1 s, two lateral white-LED-lights were turned on so the handle was illuminated, and remained illuminated until handle release.

The animal was asked to grasp the handle in dark and light conditions randomly presented. The orientation of the handle was visible during all trials performed in the light and not visible during trials in the dark (the handle was seen by the animal before starting the 20 trials block, as there were blocks of horizontal and blocks of vertical orientations). So, in the light condition the hand movement was visually-guided, whereas in the dark it was memory-guided, by the vision of handle orientation seen before the block. In both task conditions, the monkey was required to gaze at the fixation point. If fixation was broken, trials were interrupted on-line and discarded. For a detailed description of the control system of trial execution see^59^.

#### Visual control task

A control task was performed with the same setup as the Reach-to-grasp task in the light, but with the monkey not allowed to perform any arm movement (they were prevented by a door at the front of the chair blocking the hand access to the handle). In the Visual control task, the switch-off of fixation LED cued the monkey to release the home-button to receive reward. Home-button pressing started another trial. The rationale of this task was to check the visual response of the neuron to the presentation of the handle when the grasping of the handle was not required, nor performed, i.e. when the handle was not the target of a grasping action.

Video inspections of the passive viewing condition revealed that the monkey attempted to reach the handle only a few times, during the first trials of each passive viewing block. After the first trials he understood that the required task was different. In these few cases, the trials where he attempted to reach the handle were removed by the analysis.

#### Data analysis

All the analyses were performed using custom scripts in Matlab (Mathworks, Natick, MA, US).

V6A neural activity during the Reach-to-grasp task was divided into the following time epochs:

- *FREE*: from home button pressing to fixation LED light-up (this epoch was used as a reference period and was in the dark in both task conditions).
- *PLAN*: the last 500 ms before the go-signal. It includes cell discharge during arm movement preparation.
- *MOV*: from 200 ms before movement onset (home-button release) to movement end (handle pulling). It includes cell discharge during grasping execution.
- *HOLD*: from handle pulling to 200 ms before the onset of return movement (handle release). It includes cell discharge during handle pulling.V6A neural activity during the Visual control task was quantified in the following time epochs:
- *FREE*: from home button pressing to fixation LED light-up (this epoch was used as a reference period).
- *VIS*: response to handle presentation, from 40 ms after object illumination to 300 ms after it.

Only the units tested for at least 7 trials for each condition were taken into account. Indeed, only 0.51% of units in the entire population were analysed in only 7 trials. The mean number of trials analysed was 16.24 ± 7.19 per condition. Task-related cells were defined as cells significantly modulated in at least one of the epochs PLAN, MOV, HOLD with respect to FREE (Student’s t-test, p < 0.02, corrected for multiple comparisons) in at least one visual background condition (dark, light) in the Reach-to-grasp task. Only task-related cells were further analysed.

Significant modulation of neural activity by hand orientation and visual conditions of the task was studied through a two-way ANOVA (factor 1: hand orientation (2 levels, horizontal, vertical); factor 2: visual condition (2 levels, light, dark)).

To assess the strength of visual modulation on grasping activity, we calculated the following index (Visual Index):


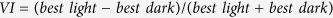


where ‘best light’ and ‘best dark’ are the mean average rates of discharge of each neuron in light and dark conditions, respectively, for the wrist orientation evoking the highest mean response in that visual condition. The index ranges from −1 to 1. A neuron whose grasping activity is elicited only in the presence of visual input (i.e. in the light) will have a value of 1, whereas a value of −1 denotes a neuron active only in the dark. Values close to 0 indicate that the neuron is modulated similarly by grasping in light and dark conditions.

Population responses of neurons modulated by visual information during grasping were computed as average spike density functions (SDFs). An SDF was calculated (Gaussian kernel, half-width 40 ms) for each neuron included in the analysis. SDF was averaged across all trials with the preferred wrist orientation, separately for light and dark conditions. The peak discharge rate of the neuron was used to normalize SDFs. The normalized SDFs were then averaged to derive population responses, and population SDFs in the dark and in the light were compared with each other using a permutation test (10,000 iterations)^25^.

To further determine neural activity patterns that may have not been captured by the above statistical techniques, we performed a principal component analysis (PCA) on the data. PCA involves computing the covariance matrix of the data with respect to all dimensions of interest, and in extracting eigenvectors and eigenvalues of this matrix. The resulting eigenvectors constitute a different set of components that, linearly summed, allow reconstruction of the data. This analysis can provide further insight for data interpretation because each eigenvalue represents the weight of the corresponding component of the data, i.e., the amount of variance explained. Therefore, once the eigenvalues are normalized, they can be ranked based on their relative importance in capturing the variance of the whole dataset. If one or more components have eigenvalues very close to 0, it means that some of the original dimensions can be computed from the others, and are thus redundant.

We applied PCA in each of the data sets (cells whose grasp-related activity is higher in the light, called the ‘excited cells’, and cells whose grasp-related activity is higher in the dark, called ‘inhibited cells’) to the activity of neurons from 800 ms before movement onset to the end of the HOLD period, so as to include all the epochs considered (DELAY, MOV and HOLD). Moreover, we applied PCA for each epoch (DELAY, MOV, HOLD) independently.

To quantify the orientation sensitivity of task-related neurons in the two task conditions, we calculated a Sensitivity Index (SI) which takes into account the magnitude of the neural response to each hand orientation in each visual condition:


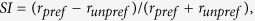


where *r*_*pref*_ is the activity for the hand orientation evoking the highest discharge and *r*_*unpref*_ is the activity for the hand orientation evoking the lowest discharge for the considered action epoch (PLAN, MOV, HOLD). The SI ranges between 0 and 1. Neurons with values near 0 show the same magnitude of response for all wrist orientations, whereas neurons with values near 1 indicate a strict selectivity for one wrist orientation. SI was calculated for each neuron and for the two visual conditions. To compare the SI of the same cell in different visual conditions, confidence intervals on the preference indices were estimated using a bootstrap test. Synthetic response profiles were created by drawing *N* firing rates (with replacement) from the *N* repetitions of experimentally determined firing rates. The SI was recomputed using these *N* firing rates. Ten thousand iterations were performed, and confidence intervals were estimated as the range that delimited 95% of the computed indices^61^. For each neuron, a synthetic mean firing rate was computed by averaging the activity of randomly resampled trials. For each configuration, this was done twice and the SI with these synthetic firing rates was calculated. This procedure was repeated 1000 times and the average value was computed. The SI values for each pair of configurations were then averaged. Using this computation, we obtained 2 values for each neuron, which were plotted against one another; they represented the noise values. Using these estimates of measurement for the population, we determined a border that encompassed 95% of the noise values (see grey line in Fig. 6A). Neurons plotted below this border (i.e. inside the grey area) had sensitivity not statistically distinguishable from zero.

By considering the neural discharge in MOV, we divided the cells into 3 categories, similarly to what was done by Murata *et al*.^19^. A neuron was classified as ‘*Visual*’ if its discharge during MOV was significantly different with respect to the discharge during FREE in the light but not in the dark (Student’s t-test, p < 0.05). A neuron was classified as ‘*Motor*’ if its discharge during MOV: i) was significantly different with respect to the discharge during FREE in both the dark and the light (Student’s t-test, p < 0.05) and ii) was similar in the dark and in the light (Student’s t-test, p > 0.05). A neuron was classified as ‘*Visuomotor*’ if its discharge during MOV: i) was significantly different with respect to the discharge during FREE in the dark, or in both the dark and the light (Student’s t-test, p < 0.05) and ii) was different between the dark and the light (Student’s t-test, p < 0.05). This categorization was performed on the condition (handle orientation) evoking the highest discharge in the light.

Movement times were calculated trial-by-trial based on behavioral markers, by subtracting the time of occurrence of home button release from the time of occurrence of handle pulling.

Responses to the handle observation were assessed by comparing neural discharge during VIS with baseline activity (FREE) by a Student’s t-test (p < 0.05).

## Additional Information

**How to cite this article**: Breveglieri, R. *et al*. Neural activity in the medial parietal area V6A while grasping with or without visual feedback. *Sci. Rep.* **6**, 28893; doi: 10.1038/srep28893 (2016).

## Figures and Tables

**Table 1:** Incidence of Visual (V), Motor (M) and Visuomotor (VM) neurons in areas V6A and AIP.

Area	Reference	V (%)	M (%)	VM (%)
V6A	Present results	**21**	**27**	**52**
AIP	19	30	26	44
AIP	18	39	13	48
AIP	17	26	33	37
AIP	**means** (±SD)	**32** (±6.66)	**24** (±10.15)	**43** (±5.57)

The categorization criteria used in the current study are the same as those used in Sakata’s and Murata’s papers for AIP^17^,^18^,^19^.
